# Measurement accuracy of prototype non-contrast, compressed sensing-based, respiratory motion-resolved whole heart cardiovascular magnetic resonance angiography for the assessment of thoracic aortic dilatation: comparison with computed tomography angiography

**DOI:** 10.1186/s12968-020-00697-x

**Published:** 2021-02-08

**Authors:** Basel Yacoub, Robert E. Stroud, Davide Piccini, U. Joseph Schoepf, John Heerfordt, Jérôme Yerly, Lorenzo Di Sopra, Jonathan D. Rollins, D. Alan Turner, Tilman Emrich, Fei Xiong, Pal Suranyi, Akos Varga-Szemes

**Affiliations:** 1 Division of Cardiovascular Imaging, Department of Radiology and Radiological Science, Medical University of South Carolina, Ashley River Tower, MSC 226, 25 Courtenay Dr, Charleston, SC 29425 USA; 2 Department of Diagnostic and Interventional Radiology, Lausanne University Hospital and University of Lausanne, Lausanne, Switzerland; 3 Advanced Clinical Imaging Technology, Siemens Healthcare AG, Lausanne, Switzerland; 4 Center for Biomedical Imaging (CIBM), Lausanne, Switzerland; 5 College of Medicine, Medical University of South Carolina, Charleston, SC USA; 6 Department of Radiology, University Medical Center of the Johannes Gutenberg University Mainz, Mainz, Germany; 7 German Center for Cardiovascular Research (DZHK), Partner Site Rhine Main, Mainz, Germany; 8 Cardiovascular MR R&D, Siemens Medical Solutions USA Inc, Charleston, SC USA

**Keywords:** Aortic dilatation, Aortic aneurysm, Magnetic resonance angiography, Compressed sensing, Computed tomography

## Abstract

**Background:**

Patients with thoracic aortic dilatation who undergo annual computed tomography angiography (CTA) are subject to repeated radiation and contrast exposure. The purpose of this study was to evaluate the feasibility of a non-contrast, respiratory motion-resolved whole-heart cardiovascular magnetic resonance angiography (CMRA) technique against reference standard CTA, for the quantitative assessment of cardiovascular anatomy and monitoring of disease progression in patients with thoracic aortic dilatation.

**Methods:**

Twenty-four patients (68.6 ± 9.8 years) with thoracic aortic dilatation prospectively underwent clinical CTA and research 1.5T CMRA between July 2017 and November 2018. Scans were repeated in 15 patients 1 year later. A prototype free-breathing 3D radial balanced steady-state free-precession whole-heart CMRA sequence was used in combination with compressed sensing-based reconstruction. Area, circumference, and diameter measurements were obtained at seven aortic levels by two experienced and two inexperienced readers. In addition, area and diameter measurements of the cardiac chambers, pulmonary arteries and pulmonary veins were also obtained. Agreement between the two modalities was assessed with intraclass correlation coefficient (ICC) analysis, Bland–Altman plots and scatter plots.

**Results:**

Area, circumference and diameter measurements on a per-level analysis showed good or excellent agreement between CTA and CMRA (ICCs > 0.84). Means of differences on Bland–Altman plots were: area 0.0 cm^2^ [− 1.7; 1.6]; circumference 1.0 mm [− 10.0; 12.0], and diameter 0.6 mm [− 2.6; 3.6]. Area and diameter measurements of the left cardiac chambers showed good agreement (ICCs > 0.80), while moderate to good agreement was observed for the right chambers (all ICCs > 0.56). Similar good to excellent inter-modality agreement was shown for the pulmonary arteries and veins (ICC range 0.79–0.93), with the exception of the left lower pulmonary vein (ICC < 0.51). Inter-reader assessment demonstrated mostly good or excellent agreement for both CTA and CMRA measurements on a per-level analysis (ICCs > 0.64). Difference in maximum aortic diameter measurements at baseline vs follow up showed excellent agreement between CMRA and CTA (ICC = 0.91).

**Conclusions:**

The radial whole-heart CMRA technique combined with respiratory motion-resolved reconstruction provides comparable anatomical measurements of the thoracic aorta and cardiac structures as the reference standard CTA. It could potentially be used to diagnose and monitor patients with thoracic aortic dilatation without exposing them to radiation or contrast media.

## Background

Thoracic aortic ectasia is defined as a localized dilatation that is less than 150% of the normal aortic diameter [1]. Such dilatation may progress into an aortic aneurysm (> 150% increase in diameter) or other potentially life-threatening conditions such as aortic rupture or dissection [1, 2]. Once thoracic aortic dilatation has been diagnosed, a “watch and wait” surveillance program is initiated until the risk of aortic rupture outweighs the potential risks of the surgical repair [3]. During this period, patients are typically examined annually with computed tomography angiography (CTA) of the chest [4]. Yet, this practice raises concerns regarding the administration of multiple doses of iodinated contrast media and repeated exposure to ionizing radiation [5, 6]. These concerns are even greater in young patients with aortic dilatation arising from genetic connective tissue disorders, such as Marfan syndrome [7]. As such, alternative imaging modalities that may reduce or even eliminate cumulative exposure to radiation and repeated contrast administration would be of great benefit to such patients.

The choice of the preferred imaging modality for the evaluation of thoracic aortic dilatation is based on patient-related factors (e.g. age, renal function, hemodynamic stability, etc.), and institutional resources. Current American Heart Association [1] and European Society of Cardiology [2] guidelines do not specify a preferred imaging modality for the assessment of non-emergent aortic disease. Despite the guidelines emphasize the need for minimizing episodic and cumulative radiation exposure, CTA often remains the method of choice due to its wide availability, speed, and isotropic spatial resolution [8].

Various potential cardiovascular magnetic resonance (CMR) techniques have been proposed for the assessment of aortic disease to avoid radiation and contrast exposure in these patients. Among these, balanced steady-state free-precession (bSSFP) based bright blood imaging is very common [9–14]. Conventional 3D bSSFP CMR angiography (CMRA) techniques require respiratory navigation, which results in unpredictable and excessively long acquisition times of up to 28 min in most cases [15, 16]. In addition, failure of respiratory gating or respiratory motion correction often results in unsuccessful acquisitions in as many as 14% of cases [16]. Several techniques have been proposed to overcome this difficulty and provide 100% scan efficiency, which in turn substantially shortens the acquisition time to 5–8 min [17–20]. Among those, respiratory self-navigation is one of the promising alternatives [21, 22], however, this technique may suffer from artifacts among other potential limitations [23–25].

A novel image reconstruction framework, extradimensional golden-angle radial sparse parallel (XD-GRASP) CMR, was recently introduced with the advantage of integrating the benefits of reduced k-space sampling and sparse reconstruction [24, 26]. Such a technique has also been used to reconstruct 3D radial golden-angle coronary artery CMRA data acquired during free-breathing at multiple respiratory phases by exploiting the sparsity along the respiratory dimension [27]. While the image quality of radial XD-GRASP CMRA has been investigated in comparison to a radial self-navigated CMRA technique before [25], its ability to accurately evaluate quantitative cardiovascular parameters and monitor disease progression remains unestablished.

Therefore the purpose of this study was to evaluate the feasibility of the non-contrast, XD-GRASP-based, respiratory motion-resolved whole-heart CMRA technique against reference standard CTA, for the quantitative assessment of cardiovascular anatomy and monitoring of disease progression in patients with thoracic aortic dilatation.

## Methods

### Patients

The study protocol was approved by our Institutional Review Board and written informed consent was obtained from every patient. All procedures were conducted in compliance with Health Insurance Portability and Accountability Act guidelines. Twenty-four patients who had undergone a clinically indicated CTA between July 2017 and November 2018 for the evaluation and follow up of their known thoracic aortic dilatation, were prospectively enrolled for a research CMRA. Our study cohort partially overlapped with the image quality cohort reported before, comparing XD-GRASP to self-navigated whole heart CMRA [25]. General CMR exclusion criteria were applied to patient selection. The research CMRA was performed within 30 days following the clinical CTA. The patients’ medical charts were accessed to obtain demographics and medical history. A subset of the patient cohort (n = 15) underwent follow up CTA and CMRA 1 year after their baseline imaging to evaluate for disease progression. Baseline and follow up scans were performed with the same imaging protocols. Follow up CTA and CMRA were acquired within 30 days.

### CTA protocol

CTA studies were conducted on a 3rd generation dual source CT system (SOMATOM Force; Siemens Healthineers, Forchheim, Germany) according to standard of care at our institution. Image acquisition was performed using prospective electrocardiographic (ECG) triggering at 70% of the R–R interval if the heart rate was < 70 bpm or 40% if the heart rate was > 70 bpm. Automated tube current modulation (CareDose, Siemens Healthineers) was utilized, with a reference tube current time product of 256 mAs per rotation, gantry rotation time 280 ms, and collimation 64 × 2 × 0.6 mm. Iodinated contrast material (Iohexol; 350 mg of organic iodine/ml, Omnipaque 350, GE Healthcare, Waukesha, Wisconsin, USA) was intravenously administered. CTA raw data were reconstructed using a standard medium-sharp (I26f) reconstruction algorithm and sinogram affirmed iterative reconstruction (Safire, strength level 3, Siemens). Images were reconstructed with 0.75 mm slice thickness at 0.3 mm increments.

### CMR protocol

A 1.5T clinical system (MAGNETOM Avanto Dot, Siemens Healthineers, Erlangen, Germany) was used to obtain the CMR scans. Patients were scanned head-first in a supine position. A multi-channel spine phased-array radiofrequency coil with 24 elements integrated into the patient table and a six element, 6-channel phased-array surface coil was used for signal reception. Acquisitions were ECG triggered.

Based on the initial scout images, a free-breathing 2D bSSFP cine image set in a parasagittal long-axis view of the left ventricle was acquired using the following parameters: repetition time/echo time (TR/TE), 2.3/1.1 ms; field of view (FOV), 340 × 340 mm; matrix, 192^2^; number of segments, 15; reconstructed phases, 25; temporal resolution, 45 ms; flip angle, 77°; number of averages, 3; and parallel acquisition acceleration factor, 2. Cine image data were used to match the timing of the whole-heart CMRA to that of CTA.

Whole-heart CMRA was performed using a prototype pulse sequence employing a 3D radial trajectory following a spiral phyllotaxis pattern [13, 27]. Image acquisition was ECG triggered and image collection was positioned during the cardiac cycle according to the phase that the CTA was reconstructed at. The typical duration of the image acquisition window was 96 ms, determined by the number of k-space lines read out per cardiac cycle (average of 32 lines, ~ 3 ms each). Typically, a total of ~ 12,000 k-space lines were read, distributed over 377 heartbeats.

**Fig. 1:**
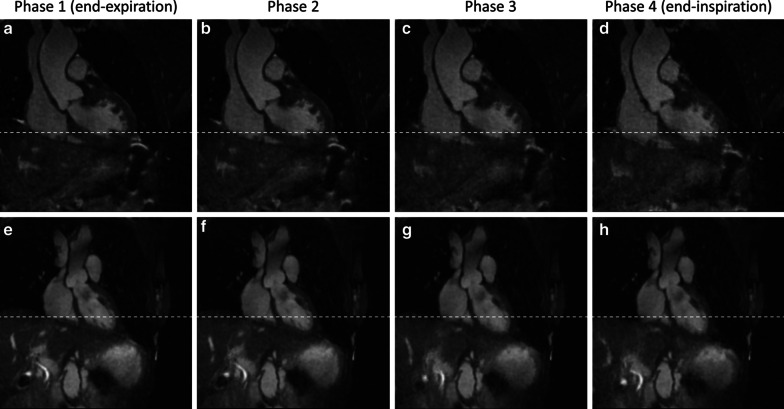
Representative case examples demonstrating image reconstruction at different respiratory phases. Coronal view images reconstructed at four different respiratory phases are shown in a 60-year-old man (**a**–**d**) and a 70-year-old woman (**e**–**h**). The reference lines (white dotted lines) indicate the top of the dome of the diaphragm at the end-inspiratory phase. Additional GIF files are provided to demonstrate respiratory motion (Additional files 1 and 2)

The following imaging parameters were used to image the entire thoracic aorta: TR/TE, 3.1/1.5 ms; FOV, (320 mm)^3^; matrix, 192^3^; isotropic voxel size, (1.66 mm)^3^; flip angle, 115°; and bandwidth, 898 Hz/pixel. Raw data were exported offline after the acquisition and then processed on a dedicated workstation using an XD-GRASP framework similar to the one previously described, fully implemented in MATLAB 2015a (MathWorks, Natick, Massachusetts, USA) [24, 28]. The signal-readouts from individual heartbeats of the 3D radial acquisition were binned according to their respiratory phase using a respiratory signal extracted directly from the imaging data [28]. The resultant series of undersampled images were then reconstructed using an XD-GRASP framework, which promotes sparsity along the respiratory dimension [26]. The respiratory phase of CMRA was matched to the respiratory phase of the corresponding CTA. Representative image examples demonstrating the different respiratory phases in two patients are shown in Fig. 1 and Additional files 1 and 2.

### Image analysis

**Fig. 2:**
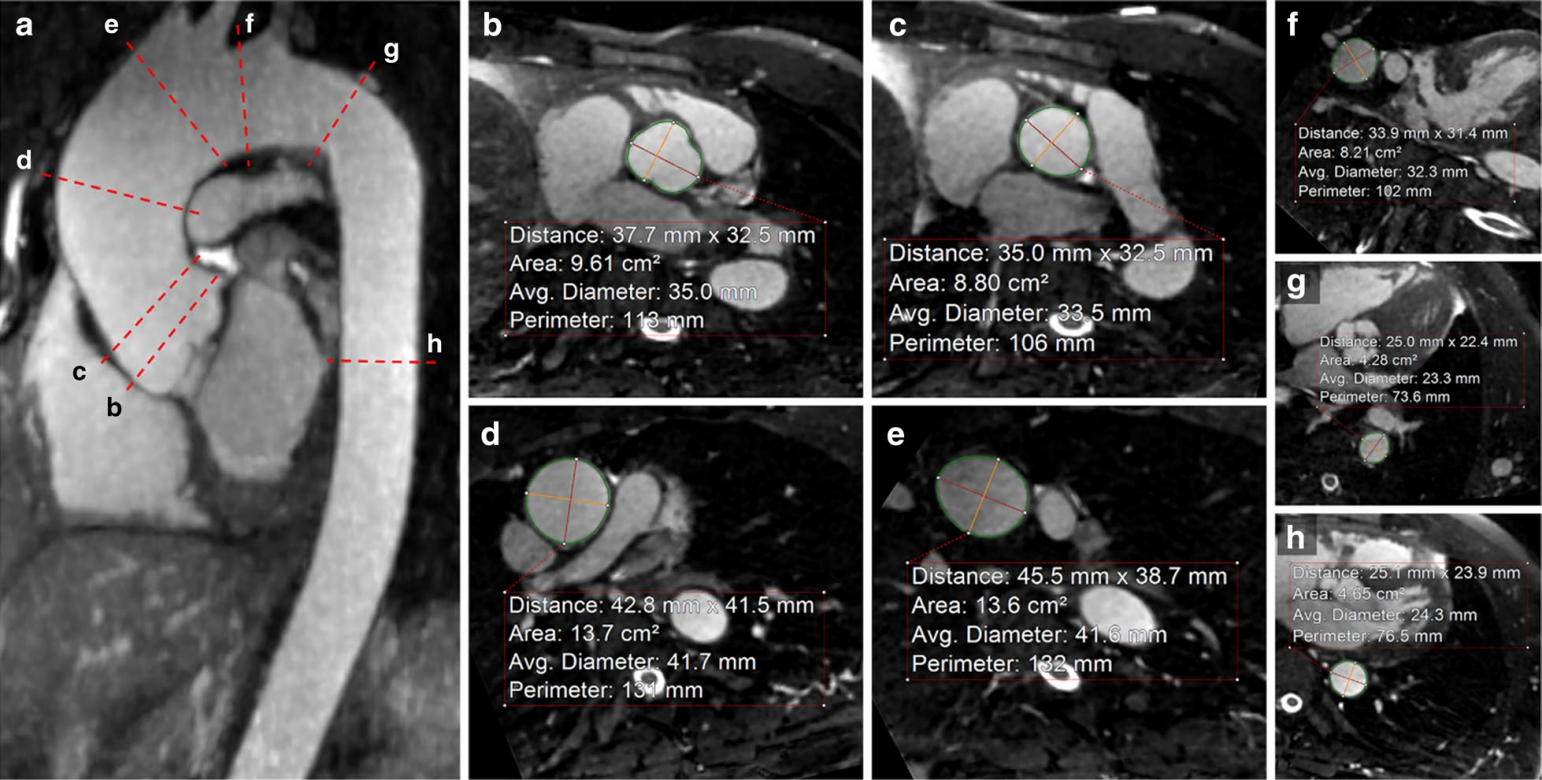
An example for the aortic measurement levels at the seven anatomical landmarks. Representative cardiovascular magnetic resonance angiography (CMRA) images in a 59-year-old man with ascending aortic dilatation. Candy cane view of the aorta (**a**) and the typical measurement planes at the level of the sinus (**b**), sinotubular junction (**c**), mid ascending aorta (**d**), proximal (**e**) and mid arch (**f**), proximal (**g**) and mid descending aorta (**h**) are shown

CTA and CMRA images were reviewed on a dedicated workstation (Aquarius iNtuition Edition v4.4.12, TeraRecon, Inc., Foster City, California, USA). Two experienced readers, with 11 and 2 years of experience respectively, and two inexperienced readers individually reviewed all scans. CTA and CMRA images were evaluated independently in a blinded fashion with a time gap of 2 weeks to minimize recall bias. Standard axial, sagittal and coronal planes were used to generate multi-planar reformats (MPR) allowing for the visualization of the aorta at each level. MPR images were used to measure area, circumference and diameter of the aorta using the double oblique technique. An automated aortic edge detection tool was utilized, and manual adjustments were performed when necessary. The aorta was assessed at the following 7 anatomical landmarks: (1) aortic sinus of Valsalva, (2) sinotubular junction, (3) mid ascending aorta (half way between (2) and (4)), (4) proximal aortic arch (by the origin of the innominate artery), (5) mid aortic arch (between left common carotid and subclavian arteries), (6) proximal descending aorta (2 cm distal to left subclavian artery) and (7) mid descending aorta (midpoint between (6) and diaphragm) [1]. In addition, the maximum diameter of the dilatation was obtained. A representative example for the measurement levels is shown in Fig. 2. To further evaluate the accuracy of the CMRA technique in more challenging measurements, for example small caliber vessels and cardiac chambers that are more sensitive to motion, the following parameters were evaluated: area and maximum diameter of the left ventricle, left atrium, right ventricle, and right atrium measured on a 4-chamber view, and area and diameter of the left and right pulmonary arteries, as well as the pulmonary veins using MPRs.

The 1-year follow up CTA and CMRA scans were analyzed by the same experienced readers in a blinded fashion, similarly as described for the baseline evaluation. The readers measured the maximum diameter of the aortic dilatation. Disease progression was evaluated by calculating the difference between the follow up and baseline scans, as well as the percentage of difference relative to the baseline scan.

### Statistical analysis

All statistical analysis was performed on SPSS (v25, Statistical Package for the Social Sciences, International Business Machines, Inc., Armonk, New York, USA). Categorical variables are described as counts with percentages and continuous variables as mean ± standard deviation (SD). Means of the measurements obtained by the experienced readers were used for inter-modality comparison. Two-way mixed effects, absolute agreement and single rater intraclass correlations (ICC) were used to assess agreement between CTA and CMRA measurements of area, circumference and diameter at each of the cardiac and vascular locations. Bland–Altman plots were used to illustrate any differences between CMRA and CTA measurements, as well as between baseline and follow up assessments. ICC was also used to assess inter-reader agreement and was interpreted as follows: < 0.5, poor agreement; 0.5–0.75, moderate agreement; 0.75–0.9, good agreement; and > 0.9, excellent agreement [29].

## Results

**Fig. 3:**
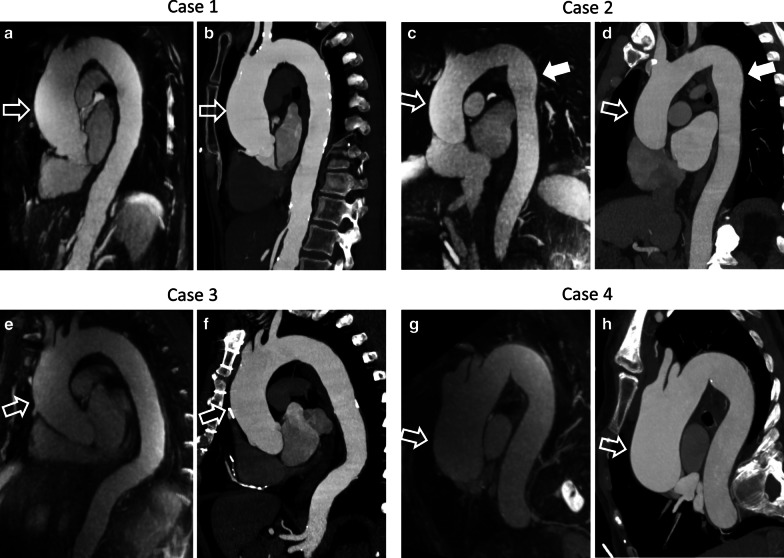
Representative case examples demonstrating comparison between CMRA and computed tomography angiography (CTA). Representative CMRA (**a**, **c**, **e** and **g**) and CTA (**b**, **d**, **f** and **h**) maximum intensity projection images displayed as 5 mm thick slabs are shown in the candy cane view of the aorta. Case 1 (**a** CMRA and **b** CTA) features a 68-year-old woman with ascending aortic dilatation (maximum diameter of 49.2 mm, open arrows). Case 2 (**c** CMRA and **d** CTA) shows a 73-year-old man with dilatation affecting both the ascending (open arrows) and the descending aorta (solid arrows) with maximum diameters of 44.0 mm and 35.8 mm, respectively. Case 3 (**e** CMRA and **f** CTA) presents a 64-year-old man with dilatation predominantly in the ascending aorta reaching a maximum of 45.3 mm (open arrows). The descending aorta has a borderline maximum diameter of 30.7 mm. Case 4 (**g** CMRA and **h** CTA) shows a 77-year-old woman with a tortuous thoracic aorta, likely accentuated by scoliosis. The maximum diameter of the ascending aorta (open arrows) is 52.6 mm

A total of 24 patients (16 males; 45 to 81 years) were enrolled. Seventeen patients had a predominantly ascending aortic dilatation while seven subjects had dilatation predominantly affecting the descending aorta. The average maximum baseline diameter of the dilatation in the ascending aorta measured by CTA and CMRA was 44.4 ± 4.2 mm and 43.7 ± 4.0 mm, respectively (ICC 0.94), while for the descending aorta was 40.3 ± 4.0 mm and 39.3 ± 4.8 mm, respectively (ICC 0.93). Detailed patients’ characteristics are reported in Table 1. Representative CTA and CMRA image examples are shown in Fig. 3.

**Table 1:** Patient characteristics

Gender (males)	16 (66.7%)
Age (years)	68.6 ± 9.8
Weight (kg)	88.3 ± 18.0
Body mass index (kg/m^2^)	28.9 ± 5.8
Body surface area (m^2^)	2.0 ± 0.2
Diabetes mellitus	5 (20.8%)
Hypertension	11 (45.8%)
Dyslipidemia	12 (50.0%)
Coronary artery disease	10 (41.7%)
Myocardial infarction	3 (12.5%)
Stroke	3 (12.5%)
Coronary artery bypass graft	2 (8.3%)

Data are displayed as mean ± standard deviation or frequency (%)

Mean area, circumference and diameter aortic measurements by the two experienced readers are reported in Table 2. ICC for level-based agreement between measurements on CTA and CMRA are also reported. ICC for area ranged from 0.90 at the sinotubular junction to 0.96 at the mid descending aorta. For circumference measurements, it ranged from 0.86 at the proximal arch to 0.97 at the mid descending aorta. For diameter measurements, it ranged from 0.84 at the proximal descending aorta to 0.97 at the mid descending aorta.

**Table 2:** Inter-modality agreement

Aorta levels	Area (cm^2^)			Circumference (mm)			Diameter (mm)		
	CTA	CMRA	ICC	CTA	CMRA	ICC	CTA	CMRA	ICC
Sinus	11.8 ± 2.8	12.0 ± 2.7	0.96	127.6 ± 14.7	130.8 ± 15.7	0.92	39.2 ± 4.9	41.0 ± 5.0	0.90
Sinotubular junction	10.4 ± 2.0	10.4 ± 2.0	0.90	115.5 ± 11.1	116.7 ± 12.1	0.90	36.5 ± 3.5	37.0 ± 3.8	0.89
Mid ascending aorta	14.1 ± 2.6	14.2 ± 2.5	0.94	133.5 ± 13.7	135.7 ± 12.9	0.90	42.3 ± 4.2	43.1 ± 4.2	0.93
Proximal arch	11.4 ± 2.6	11.6 ± 2.7	0.93	120.5 ± 14.3	123.0 ± 14.7	0.86	38.1 ± 4.5	39.1 ± 4.8	0.91
Mid arch	7.7 ± 2.0	7.3 ± 2.0	0.91	98.6 ± 12.4	97.6 ± 13.0	0.94	31.3 ± 4.0	31.1 ± 4.0	0.94
Proximal descending aorta	6.2 ± 1.3	6.0 ± 1.2	0.92	89.0 ± 9.7	88.9 ± 9.1	0.90	28.1 ± 3.1	28.1 ± 3.0	0.84
Mid descending aorta	6.2 ± 3.2	6.1 ± 2.6	0.96	88.0 ± 19.5	87.6 ± 16.7	0.97	27.8 ± 6.2	28.1 ± 5.5	0.97

Area, circumference and diameter measurements on CTA and CMRA at different levels of the thoracic aorta. Data are reported as means with standard deviation. Inter-modality agreement is shown with ICC values

*CTA* computed tomography angiography, *CMRA* cardiovascular magnetic resonance angiography, *ICC* intra-class correlation coefficient

**Fig. 4:**
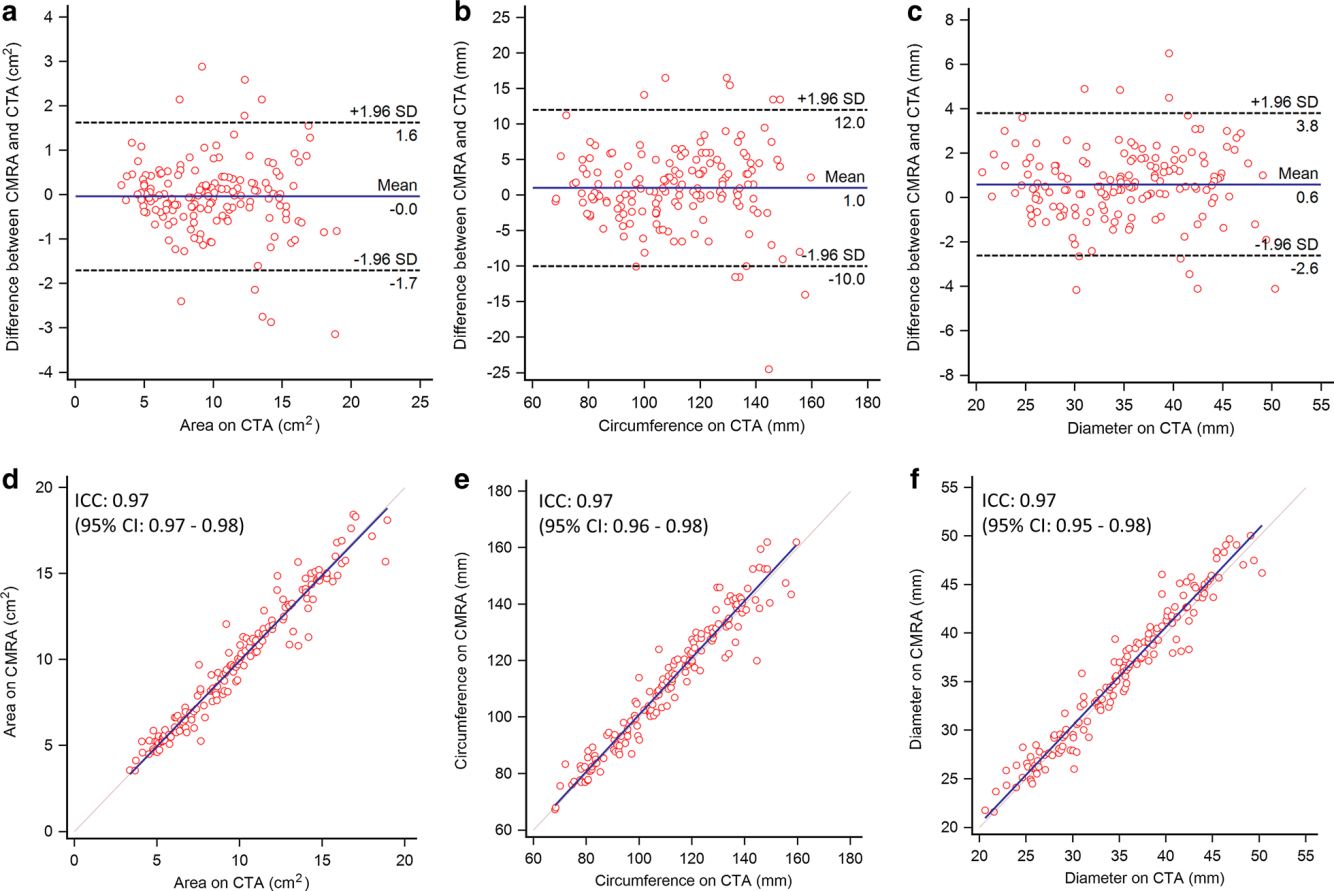
Bland–Altman and scatter plots comparing baseline CTA and CMRA measurements. Bland–Altman plots (**a**–**c**) show high agreement between CTA and CMRA for the measurements of aortic anatomic parameters. The means of differences (solid line) shown by the Bland–Altman plots are 0 cm^2^ (area), 1.0 mm (circumference), and 0.6 mm (diameter). Dashed lines show the 95% limits of agreement (± 1.96 standard deviation). Scatter plots (**d**–**f**) also show excellent agreement between measurements on CTA and CMRA with ICC values for area, circumference and diameter of 0.97 for each. *CTA* computed tomography angiography, *CMRA* cardiovascular magnetic resonance angiography, *ICC* intra-class correlation coefficient

Area and diameter measurements of the left cardiac chambers showed similarly good levels of agreement with all ICCs > 0.80, while agreement for the right chambers was moderate to good (all ICCs > 0.56). Pulmonary artery and pulmonary vein area and diameter measurements showed good to excellent inter-modality agreement (ICC range 0.79–0.93), except for the left lower pulmonary vein, for which only poor to moderate agreement was achieved (ICC < 0.51). Further details are reported in Table 3. The means of differences in area, circumference and diameter measurements between CTA and CMRA were 0.0 cm^2^, 1.0 mm and 0.6 mm, respectively. ICC for agreement of all area, circumference and diameter measurements were 0.97 for each. Bland–Altman plots and scatter plots for all area, circumference and diameter measurements are illustrated in Fig. 4.

**Table 3:** Inter-modality agreement

Structures	Area (cm^2^)			Diameter (mm)		
	CTA	CMRA	ICC	CTA	CMRA	ICC
Left ventricle	35.5 ± 6.9	33.5 ± 6.2	0.80	47.9 ± 7.4	46.7 ± 6.7	0.86
Left atrium	19.9 ± 5.5	18.9 ± 5.2	0.90	52.2 ± 8.4	50.8 ± 7.5	0.85
Right ventricle	29.0 ± 7.3	29.2 ± 7.2	0.84	49.4 ± 8.6	48.2 ± 8.5	0.76
Right atrium	16.8 ± 4.8	17.6 ± 4.1	0.84	40.2 ± 6.8	43.8 ± 5.2	0.56
Left pulmonary artery	5.1 ± 1.2	4.8 ± 1.1	0.91	25.2 ± 2.9	24.5 ± 2.8	0.91
Right pulmonary artery	4.7 ± 1.2	4.3 ± 1.1	0.84	24.2 ± 3.3	23.2 ± 3.0	0.86
Left lower pulmonary vein	1.7 ± 0.5	1.8 ± 0.6	0.51	14.5 ± 2.2	15.1 ± 2.2	0.49
Left upper pulmonary vein	2.2 ± 0.7	2.0 ± 0.6	0.86	16.6 ± 2.5	15.9 ± 2.3	0.84
Left common pulmonary vein^a^	4.7 ± 1.1	4.5 ± 1.0	0.92	24.3 ± 2.8	23.9 ± 2.5	0.93
Right lower pulmonary vein	2.2 ± 0.9	2.3 ± 1.0	0.89	16.5 ± 3.6	16.9 ± 3.8	0.90
Right upper pulmonary vein	2.6 ± 1.0	2.7 ± 0.9	0.85	18.0 ± 3.3	18.1 ± 3.1	0.79

Area and diameter measurements of the cardiac chambers, pulmonary arteries and pulmonary veins on CTA and CMRA. Data are reported as means with standard deviation. Inter-modality agreement is shown with ICC values

^a^Left common pulmonary vein was present in six patients

Inter-reader agreement among all four readers on a per-level based assessment showed mostly moderate to excellent agreement on both CTA (ICCs for area ≥ 0.70; circumference ≥ 0.72; and diameter ≥ 0.64) and CMRA (ICCs for area ≥ 0.76; circumference ≥ 0.70; and diameter ≥ 0.72) measurements. Inter-reader agreement on CMRA measurements at each level was reflective of their agreement on the CTA measurements (Table 4).

**Table 4:** Inter-reader agreement in the aorta anatomic measurements among all four readers is shown with ICC values

Aorta levels	Area		Circumference		Diameter	
	CTA	CMRA	CTA	CMRA	CTA	CMRA
Sinus	0.92	0.89	0.83	0.77	0.77	0.78
Sinotubular junction	0.80	0.76	0.72	0.70	0.81	0.78
Mid ascending aorta	0.92	0.79	0.85	0.73	0.93	0.85
Proximal arch	0.96	0.81	0.93	0.71	0.92	0.86
Mid arch	0.90	0.80	0.88	0.75	0.90	0.79
Proximal descending aorta	0.70	0.79	0.73	0.75	0.64	0.72
Mid descending aorta	0.89	0.93	0.87	0.90	0.86	0.92

**Fig. 5:**
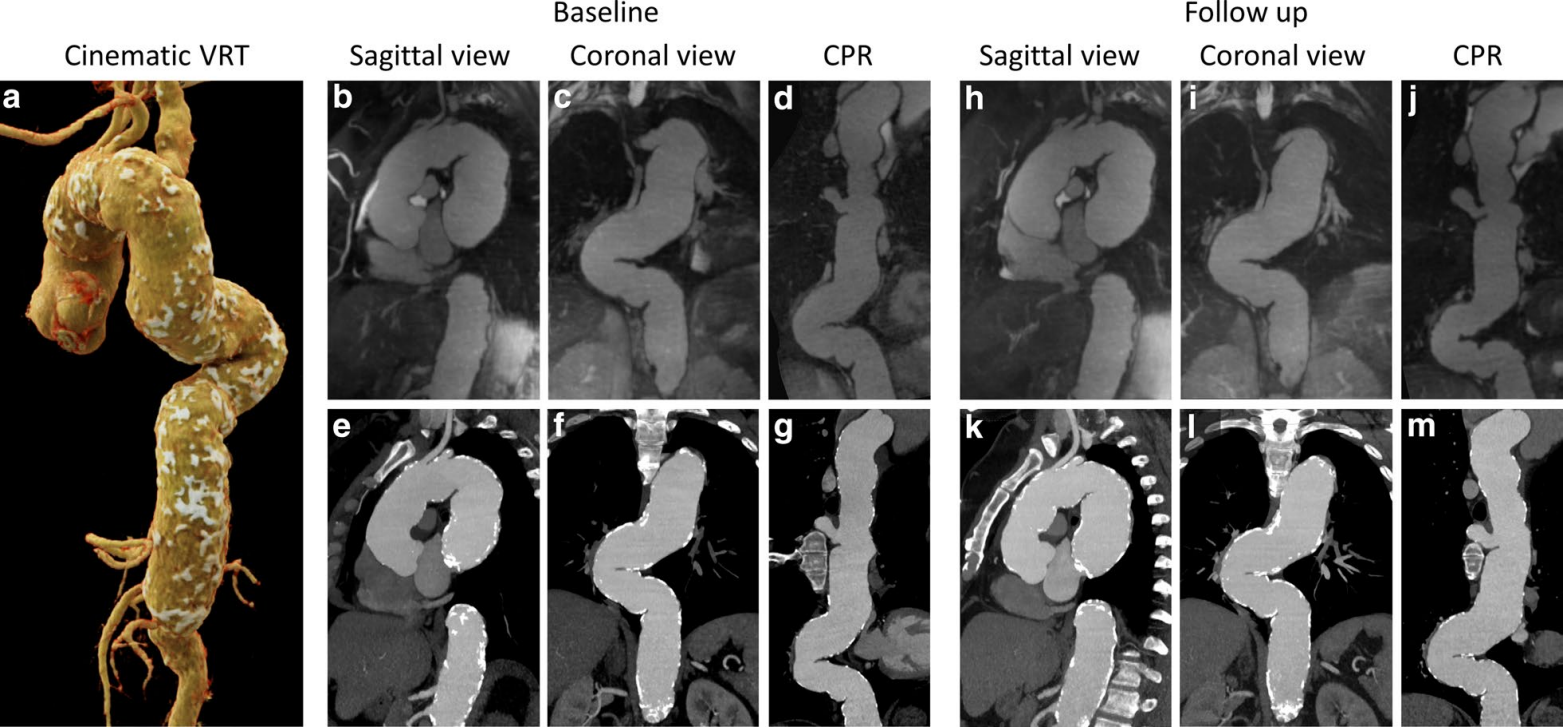
Representative case example demonstrating disease monitoring over a 1-year follow up. Cinematic volume rendering technique (**a**), generated from the baseline CTA of a 46-year-old woman, shows the overview of an extensively calcified and tortuous aorta. Aneurysmal aortic dilatation is present at the level of the root, ascending and descending aorta, extending to the level of the celiac artery. Corresponding CMRA (top row, **b**–**d** and **h**–**j**) and CTA (bottom row, **e**–**g** and **k**–**m**) images in sagittal, coronal and curved planar reformat views are shown at baseline (**b**–**g**) and at 1-year follow up (**h**–**m**). The maximum diameter increased from 47.4 to 53.0 mm (11.8%) in the ascending aorta, and from 53.2 to 54.3 mm (2.1%) in the descending aorta. *VRT* volume rendering technique, *CPR* curved planar reformat

**Fig. 6:**
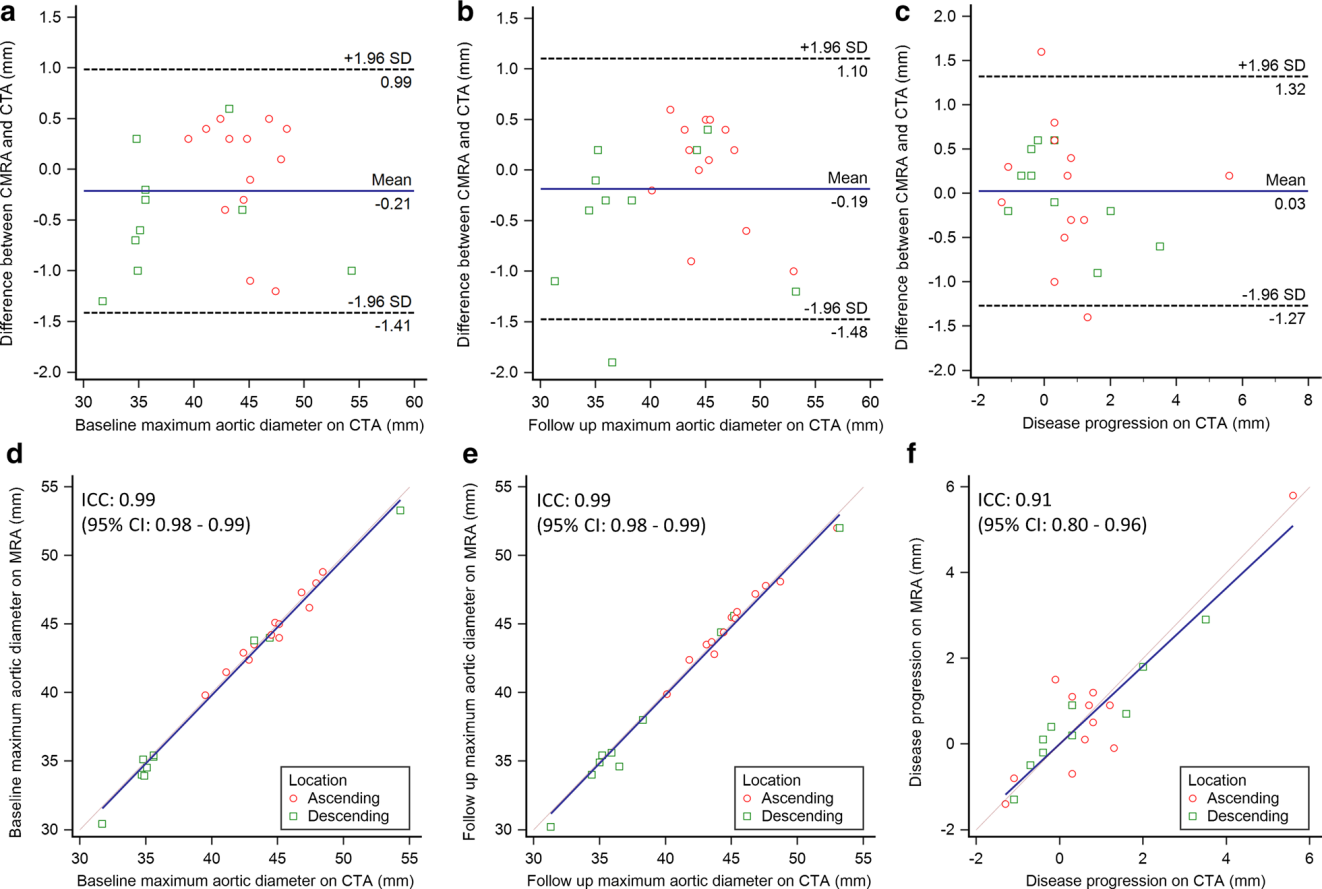
Bland–Altman and scatter plots comparing maximum aortic measurements and disease progression between CTA and CMRA. Bland–Altman plots (**a**–**c**) show good agreement between measurements of maximum aortic diameters obtained by CTA and CMRA both at baseline (mean difference: − 0.21 mm; **a**) and at 1-year follow up (mean difference: − 0.19 mm; **b**). The mean of differences for disease progression was 0.03 mm between the techniques (**c**). Scatter plots (**d**–**f**) demonstrate excellent agreement between measurements on CTA and CMRA of the baseline (**d**) and follow up (**e**) maximum aortic diameters with ICC values of 0.99. The difference between the baseline and follow up measurements, representing disease progression, showed an ICC value of 0.91 (**f**). Note, that both the ascending and descending sections of the aorta showed dilatation in some patients, therefore the number of data points displayed (n = 23) is higher than the number of subjects included (n = 15). Red markers represent the ascending, while green markers show the descending aorta. *CTA* computed tomography angiography, *CMRA* cardiovascular magnetic resonance angiography, *ICC* intra-class correlation coefficient

Out of the 24 patients, six patients chose not to take part in the follow up study and three subjects underwent aortic surgery. The remaining 15 patients (12 males) underwent follow up imaging 1 year after the baseline evaluation. Two patients showed clinically significant disease progression with a maximum diameter increase of 5.6 mm (11.8%) in the ascending aorta (baseline and follow up CMRA and CTA are shown in Fig. 5) and 3.5 mm (10.1%) in the descending aorta. The maximum aortic diameters and disease progression in this subset of patients are shown in Table 5 and Fig. 6. Excellent agreement (all ICCs > 0.9) was observed between CTA and CMRA follow up scans for the measurement of maximum aortic diameters. Agreement in progression was good to excellent for the ascending aorta, while excellent for the descending aorta.

**Table 5:** Maximum aortic diameter measurements and disease progression in the follow up cohort (n = 15)

Parameters	Baseline			1-year follow up		
	CTA	CMRA	ICC	CTA	CMRA	ICC
Ascending aorta dilatation						
Maximum diameter (mm)	44.5 ± 2.7	44.5 ± 2.6	0.98	45.3 ± 3.3	45.3 ± 3.1	0.99
Progression (mm)				0.7 ± 1.7	0.8 ± 1.8	0.90
Progression (%)				1.6 ± 3.5	1.7 ± 3.8	0.89
Descending aorta dilatation						
Maximum diameter (mm)	38.4 ± 6.8	38.0 ± 6.9	0.99	38.9 ± 6.6	38.5 ± 6.7	0.99
Progression (mm)				0.5 ± 1.4	0.5 ± 1.2	0.93
Progression (%)				1.4 ± 3.9	1.4 ± 3.1	0.93
Combined^a^						
Maximum diameter (mm)	41.9 ± 5.7	41.7 ± 5.8	0.99	42.5 ± 5.8	42.3 ± 5.9	0.99
Progression (mm)				0.6 ± 1.5	0.7 ± 1.5	0.91
Progression (%)				1.5 ± 3.6	1.6 ± 3.4	0.90

^a^Includes both predominantly ascending and descending dilatations

## Discussion

This study aimed to evaluate a recently developed non-contrast 3D radial free-breathing whole-heart CMRA acquisition and respiratory motion-resolved reconstruction technique for the assessment and monitoring of thoracic cardiovascular anatomy in patients with known thoracic aortic dilatation in comparison with reference CTA. Area, circumference, and diameter along different levels of the thoracic aorta were measured for both the CTA and CMRA according to current guidelines [1]. Overall, we found good to excellent agreement between the CTA and CMRA measurements and mostly good and excellent agreement among the experienced and inexperienced readers. Furthermore, CMRA provided excellent agreement with CTA for the monitoring of disease progression in a 1-year follow up period. These findings support that such a CMRA technique is a potential radiation- and contrast-free alternative modality to CTA for the diagnosis and monitoring of patients with thoracic aortic dilatation.

In this study, we reported good to excellent agreement on a level-based area, circumference, and diameter measurements between CMRA and CTA. Such a study design can be considered unique due to the lack of studies reported in the literature that demonstrate the ability of any whole heart CMRA technique to monitor disease progression, especially with reference CTA, the most commonly used imaging modality for the routine assessment of patients with thoracic aortic dilatation. The majority of studies published in similar patient populations used other CMR/CMRA techniques, such as contrast enhanced CMRA, 2D T2-weighted black blood or cine bSSFP for comparison [1, 12, 30–33]. A very limited number of studies investigated 3D CMRA for the evaluation of thoracic aorta (mainly the aortic root anatomy) in comparison with CTA. Ruile et al. studied patients with aortic valve stenosis prior to transcatheter aortic valve replacement and found that CMRA using a respiratory navigated 3D gradient echo fast low-angle shot (FLASH) technique allowed reliable assessment of the aortic annulus dimensions compared to CTA reference [34]. All of these studies used respiratory navigation to avoid breathing artifacts which, as mentioned in the introduction, may come with unreasonably long and unpredictable image acquisition time and a non-negligible failure rate [30–34]. Although respiratory self-navigation may sufficiently reduce acquisition time, the 1D nature of the superior-inferior self-navigation has its own shortcomings [12, 24, 25]. The novelty in the respiratory motion-resolved XD-GRASP reconstruction is that the image data can be acquired in a free-breathing fashion without the need for any kind of navigation or motion correction [24, 28]. The reconstruction algorithm extracts the respiratory motion directly from the imaging data and takes it into account as an additional dimension, without imposing a specific motion model for the reconstruction. This also allows for the selection of the most suitable phase from the respiratory domain during the post-processing steps.

While measurement accuracy is one aspect that is important when evaluating a potentially new technique for a new indication, reproducibility of the anatomic measurements is another crucial factor. As we have shown in our study, the inter-reader agreement, between the experienced and inexperienced readers showed mostly good and excellent agreement on the anatomical level-based analysis of aortic parameters for CMRA. The inter-reader agreement on CMRA was reflective of that on CTA meaning that the differences are mostly due to inherent reader differences rather than due to difficulty in reading the new CMRA technique. These results suggest that the measurements obtained from the CMRA technique are just as intuitive as measurements made from CTA and do not need extensive cardiovascular imaging experience.

A subset of our patients underwent 1-year follow up CTA and CMRA to evaluate for disease progression. Our results indicate that monitoring of change in maximum aortic diameter can be performed using the proposed CMRA technique with excellent agreement with CTA. Both CMRA and CTA were able to identify the two patients who had clinically significant disease progression and exclude aneurysm growth in the other 13 patients. Demonstrating the ability to accurately monitor disease progression further increases the value of CMRA and its potential to replace CTA, the most frequently used technique for annual follow up examinations in patients with thoracic aortic disease.

Thoracic aortic dilatation, including ectasia and aneurysm, is typically an asymptomatic process that results in a weakened aortic wall, leading to cardiovascular complications such as rupture or dissection and possible death. Thus, the importance of early detection and techniques to monitor the progression of this disease, whether acquired or genetic, is vital to these individuals. Currently, echocardiography, CTA, and CMR are the only noninvasive methods used to detect and monitor thoracic aortic dilatation [1, 35]. Transthoracic echocardiography is widely available to evaluate cardiovascular anatomy; however, its limited acoustic window is not suitable to assess the entire thoracic aorta and it is not recommended for external aortic diameter size measurements [1, 36]. The advantages of CT imaging, the current reference standard to assess thoracic aorta anatomy, include widespread availability and fast image acquisition time. In addition, CTA has been demonstrated to have a high accuracy (92%) for diagnosing thoracic aortic abnormalities [1]. However, CTA exposes patients to cumulative ionizing radiation and iodinated contrast media during annual follow up examinations [6, 37]. While a large variety of CMR techniques have been investigated to detect thoracic aortic dilatation, most of these conventional approaches have certain limitations preventing them to compete with CTA [38]. Such limitations include the need for breath-holds, the administration of gadolinium-based contrast, the extensively long table time and/or the use of 2D imaging techniques that are less suitable to visualize complex anatomy.

The prototype free-breathing whole-heart CMRA technique that we evaluated in this work can address all of these limitations as it eliminates the need for breath-holds or respiratory navigation thanks to the respiratory motion-resolved XD-GRASP reconstruction, and provides a 3D volume of the chest in a relatively short (~ 6 min) acquisition time without the use of contrast agents.

There are a few promising recent CMR pulse sequence developments that have similarly been able to address the limitations of respiratory-navigation and provide adequate image quality for the evaluation of thoracic cardiovascular anatomy. Haji-Valizadeh et al. introduced a stack-of-stars k-space sampling-based GRASP technique for self-navigated aorta CMRA and demonstrated clinically acceptable image quality compared to contrast enhanced CMRA [39]. The XD-ORCCA (Optimized Respiratory-resolved Cartesian Coronary CMR Angiography) technique by Correia et al. has been shown to provide robust respiratory-resolved motion compensation using a Cartesian approach [40]. In addition, not motion-resolved, but highly advanced motion corrected techniques with 100% respiratory efficiency and isotropic sub-millimeter resolution, such as the water/fat CMRA and the low-rank patch-based undersampled reconstruction (3D-PROST), have been found feasible for coronary CMRA [41, 42]. As a future outlook, it is worth to mention that the XD-GRASP technique has further potentials. Feng et al. reported the use of a 5D untriggered XD-GRASP technique that provides continuous acquisition with respiratory and cardiac motion resolved reconstruction [43], which has also been implemented in a free-running, fully automated and self-gated framework [28].

Our study has some limitations to consider. Sample size is relatively small, and all subjects had known thoracic aortic dilatation; however, the study was still statistically well powered for the feasibility evaluation that we proposed. Going forward, we are planning to expand the assessment of diagnostic accuracy, which may require a larger population. Moreover, we did not compare the proposed CMRA technique to the widely available Cartesian respiratory navigator gated CMRA method as CTA, an independent reference standard, was available in our patients. A previous study in healthy subjects investigating coronary arteries demonstrated signal homogeneity and time efficiency with the self-navigated technique, but inferior vessel sharpness [44]. Although, no vessel sharpness quantification was performed in the current study, we also did not visually observe limited sharpness when evaluating the aorta. Another noteworthy limitation is the time and computational power needed for image reconstruction. Currently the XD-GRASP reconstruction process is rather time consuming as the iterative algorithm requires several non-uniform Fourier transforms to be performed and the processing time may vary between 10 and 30 min based on the workstation configuration available and reconstruction settings used. However, with continued technological improvement both in computer hardware and software, this is not likely to be a long-term obstacle.

## Conclusions

The free-breathing, whole-heart CMRA technique combined with respiratory motion-resolved reconstruction provides comparable anatomical measurements of the thoracic aorta to the reference standard CTA. Thus, this novel and unique CMRA technique is a potential radiation- and contrast-free alternative modality for diagnosing and monitoring patients with thoracic aortic dilatation.

## Supplementary Information

**Additional file 1.** Demonstration of respiratory motion in transverse, coronal and sagittal views (identical to patient shown in Fig. 1, top row).

**Additional file 2.** Demonstration of respiratory motion in transverse, coronal and sagittal views (identical to patient shown in Fig. 1, bottom row).

## Data Availability

The datasets used and/or analyzed during the current study are available from the corresponding author on reasonable request.

## Abbreviations

bSSFP: Balanced steady-state free-precession
CMR: Cardiovascular magnetic resonance
CMRA: Cardiovascular magnetic resonance angiography
CPR: Curved planar reformat
CTA: Computed tomography angiography
ECG: Electrocardiogram
FLASH: Fast low angle shot
FOV: Field of view
ICC: Intra-class correlation coefficient
MPR: Multi-planar reformat
TE: Echo Time
TR: Repetition time
VT: Volume rendering technique
XD-GRASP: Extradimensional golden-angle radial sparse parallel
