# Supplementary figures and images for: Measurement accuracy of prototype non-contrast, compressed sensing-based, respiratory motion-resolved whole heart cardiovascular magnetic resonance angiography for the assessment of thoracic aortic dilatation: comparison with computed tomography angiography
Source: J Cardiovasc Magn Reson. 2021 Feb 8;23:7. doi: 10.1186/s12968-020-00697-x (PMC7871614; doi:10.1186/s12968-020-00697-x)

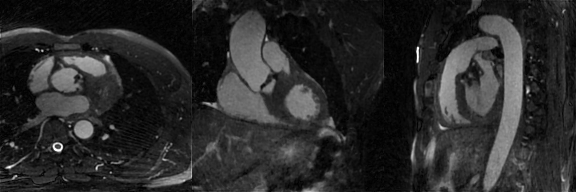

Supplement: Supplementary file 1 — Additional file 1. Demonstration of respiratory motion in transverse, coronal and sagittal views (identical to patient shown in Fig. 1, top row). [file 12968_2020_697_MOESM1_ESM.gif]

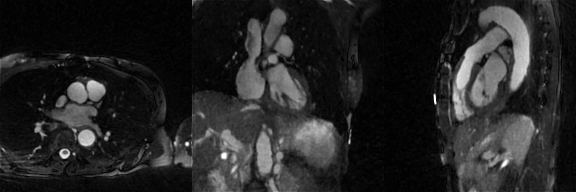

Supplement: Supplementary file 2 — Additional file 2. Demonstration of respiratory motion in transverse, coronal and sagittal views (identical to patient shown in Fig. 1, bottom row). [file 12968_2020_697_MOESM2_ESM.gif]
